# Predictive Modeling of Hospital Readmission of Schizophrenic Patients in a Spanish Region Combining Particle Swarm Optimization and Machine Learning Algorithms

**DOI:** 10.3390/biomimetics9120752

**Published:** 2024-12-11

**Authors:** Susel Góngora Alonso, Isabel Herrera Montano, Isabel De la Torre Díez, Manuel Franco-Martín, Mohammed Amoon, Jesús-Angel Román-Gallego, María-Luisa Pérez-Delgado

**Affiliations:** 1 Department of Signal Theory and Communications, and Telematics Engineering, University of Valladolid, Paseo de Belén, 15, 47011 Valladolid, Spain; isabel.herrera.montano@uva.es (I.H.M.); isator@tel.uva.es (I.D.l.T.D.); 2 Psychiatry Service, Zamora Hospital, 49021 Zamora, Spain; mfrancom@saludcastillayleon.es; 3 Personality, Evaluation and Psychological Treatments Department, University of Salamanca, Av. de la Merced, 109, 37005 Salamanca, Spain; 4 Psychosciences Research Group, Institute of Biomedicine of Salamanca (IBSAL), Paseo de San Vicente, 58-182, 37007 Salamanca, Spain; 5 Department of Computer Science, Community College, King Saud University, P.O. Box 28095, Riyadh 11437, Saudi Arabia; mamoon@ksu.edu.sa; 6 Escuela Politécnica Superior de Zamora, Universidad de Salamanca, Avda, Requejo 33, 49022 Zamora, Spain; zjarg@usal.es (J.-A.R.-G.); mlperez@usal.es (M.-L.P.-D.)

**Keywords:** machine learning, readmission, particle swarm optimization, schizophrenia

## Abstract

Readmissions are an indicator of hospital care quality; a high readmission rate is associated with adverse outcomes. This leads to an increase in healthcare costs and quality of life for patients. Developing predictive models for hospital readmissions provides opportunities to select treatments and implement preventive measures. The aim of this study is to develop predictive models for the readmission risk of patients with schizophrenia, combining the particle swarm optimization (PSO) algorithm with machine learning classification algorithms. The database used in the study includes a total of 6089 readmission records of patients with schizophrenia. These records were collected from 11 public hospitals in Castilla and León, Spain, in the period 2005–2015. The results of the study show that the Random Forest algorithm combined with PSO achieved the best results across the evaluated performance metrics: *AUC* = 0.860, *recall* = 0.959, *accuracy* = 0.844, and *F1-score* = 0.907. The development of these new models contributes to -improving patient care. Additionally, they enable preventive measures to reduce costs in healthcare systems.

## 1. Introduction

Schizophrenia is a mental disorder affecting approximately 0.75% of the global population. The symptoms that most affect people with schizophrenia are delusions, hallucinations, and disorders of behavior, feelings, and thinking [1]. Consequently, people with schizophrenia face an increased risk of mortality, suicide, and substance abuse. Hospitalization rates for people with this disorder range from 20 to 40% annually, resulting in a high social, health, economic and occupational burden [2,3]. In studies [4,5,6], risk factors such as substance abuse, the number of previous hospitalizations, age, noncompliance with medical treatment, and lack of family and social support have been identified in patients with schizophrenia who are readmitted.

In Spain, the number of hospitalizations for patients with schizophrenia reached 33,990 hospitalizations in 2022, the data from Castilla y León represent 4.35% of the total [7]. An increase in hospital readmission in mental health services directly affects the quality of patient care and increases hospitalization costs [8]. Consequently, this research aims to identify the risk factors associated with the readmission of patients with schizophrenia in the Castilla and León regions.

Currently, ML techniques are used to explore and analyze data patterns using artificial intelligence and statistical methods [9,10]. They enable the development of predictive models to analyze, learn, and generalize data behavior [11,12]. In this sense, these methods help in decision-making, diagnosis, and treatment applied in patients with schizophrenia [13]. Population-based metaheuristic algorithms such as PSO improve the convergence rate of algorithms by providing a balance between diversification and intensification during the selection of optimal solutions [14]. In this sense, the combination of optimization algorithms such as PSO with ML classification algorithms is used to improve predictive models focused on the diagnosis, hospitalization, and treatment of patients with mental health disorders.

Mental health studies such as [15,16], present a model of Random Forest (RF) with PSO. In [15], the authors address the problem of classifying people’s attention levels, while in [16] the authors develop a method of psychological crisis alert in university students. In [17], the PSO algorithm is used to eliminate redundant features and reduce the computational time for classification in the diagnosis of Alzheimer’s disease. It is also used in studies such as [18] to optimize stress prediction based on electrocardiogram signals. Focused on patients with schizophrenia, the study [13], uses PSO combined with ML techniques to develop a tool for schizophrenia detection using vocal patterns. In this sense, the studies demonstrate the improvement of ML models by applying PSO.

With respect to studies focused on the prediction of readmission, in [19] the authors developed a clinical monitoring support system for intensive care unit readmission based on PSO and ML algorithms. In studies such as [20,21], the authors develop models for predicting hospital readmissions by combining PSO with ML. Consequently, although there are related studies that combine PSO with ML to apply them in the prediction of hospital readmission, no study specifically focused on patients with schizophrenia has been found. Therefore, the aim of this study is to develop predictive models for the risk of readmissions of patients with schizophrenia in the Castilla and León region, combining the PSO algorithm with classification algorithms such as Multi-Layer Perceptron (MLP), RF, and Support Vector Machine with Radial Basis Function Kernel (SVM). In this sense, the main contributions of this study are focused on (1) the development of an optimized RF-PSO model that outperforms the performance metrics of the previous research [4], with an AUC = 0.860 and an accuracy = 84.4%. (2) Proposal of a reproducible methodological framework for the integration of metaheuristic techniques in machine learning models, with potential for application in other clinical contexts.

The methodology used in this study is described below. Subsequently, the main results obtained and a discussion of them are presented. Finally, the limitations and conclusions of this research study are presented.

## 2. Materials and Methods

### 2.1. Data Description

The study database consists of 3065 patients (6089 electronic admission records) with schizophrenia disorders. The electronic admission records of these patients were collected from the 11 public hospitals in the region of Castilla and León, Spain, between 2005 and 2015.

This database has been approved by the Ethics Committee of the “University of Valladolid”, Spain (PI 20-1780). The data included are those established in the minimum basic data set at hospital discharge [22]. The database contains inpatient diagnoses and procedures, inpatient episode characteristics, and demographic information. These data are administrative and general patient information without specific details on the patient’s clinical psychopathology (See Table S1).

### 2.2. Outcome Variable and Predictors

The variables included in this study are the result of the data set obtained in the preprocessing stage of the previous research [4]. In the previous study, six models were developed for the prediction of the risk of readmissions of patients with schizophrenia in Castilla and León region, using different ML algorithms. Consequently, the 22 predictive variables obtained in that study [4] are the ones used in the data set of this research. The outcome variable was created from the number of hospitalizations for each patient.

Based on the results obtained in the previous research, a second stage of data preprocessing was carried out. In this second stage, variables with a variance close to zero were excluded, and binarization was applied to the remaining qualitative variables. In this sense, diagnosis and procedure variables with a high proportion of constant values (>70% null values) were excluded: skin and subcutaneous tissue diseases, infectious diseases, endocrine diseases, respiratory system diseases, circulatory system diseases, digestive system diseases, genitourinary system diseases, lesions and poisoning, symptoms, signs, and poorly defined conditions, osteomioarticular system diseases, blood diseases, and the procedure Proc3. Therefore, the binarization of the quantitative variables resulted in a set of 249 independent variables.

### 2.3. Statistical Analysis

In this research, different statistical tests were used to evaluate the normal distribution of the data and homoscedasticity (equality of variances). To evaluate the normality of each of the study variables, the Lilliefors test was applied, and the Levene test was used to assess homoscedasticity. It was observed that the variables are not homoscedastic and are not normally distributed. Consequently, nonparametric tests were used to establish statistically significant differences between groups. The association between readmission and predictor variables was analyzed using the Mann–Whitney U and $X^{2}$ (Chi-square) test for quantitative and qualitative variables, respectively. A *p*-value = 0.005 was established to evaluate significant differences.

### 2.4. Machine Learning and Optimization Algorithms

#### 2.4.1. Random Forest

RF is a machine learning algorithm that combines the results of different decision trees, resulting in a single prediction [23]. RF has a great ability to analyze the classification features of complex and multidimensional data through fast learning speed, which is also robust to data sets with noise and missing values.

RF is an algorithm that contains a set of tree-structured classifiers {*h* (*x*, $\theta_{j}$), *j* = 1, … *N*}, {$\theta_{j}$} are independently distributed random vectors. The algorithm generates several trees, where each tree will cast a unit vote considering the most popular class in the input *x* [23]. As the trees are constructed, binary cuts are performed, where *j* features are randomly selected from the total *pv* predictor variables (*j* < *pv*). The node *d* is calculated through the best split point between the *j* features, and *d* is divided into child nodes through the best split.

This algorithm depends on two parameters: the tree number that makes up the algorithm and the number of *pv* variables that have been selected at each node. The setting parameter of the randomly selected predictor number (called *mtry*) does not change, while the tree type changes in the algorithm. The author [23] recommends adjusting the value of *mtry* to the square root of predictor numbers in classification problems.

#### 2.4.2. Multi-Layer Perceptron

MLP algorithm is a multilayer neural network [24]. Its architecture is composed of several interconnected layers in which each layer contains nodes. Each of the neurons is connected to neurons in the next layer and the previous layer. Network models can learn almost any pattern from the combination of nonlinear activation functions and multiple hidden layers. The nodes of the output layer represent the class labels set present in the training data set. The layer interconnections are implemented as weight matrices. These matrices are responsible for learning the latent representations of the input data through nonlinear transformations [25]. Equation (1) shows how the node in the hidden layers is computed:

(1) $$h_{1j}=f\left(\sum_{i=1}^{n}w_{ij}x_{i}+b_{j}\right)$$

where $h_{1j}\;$ is defined as node *j* of the hidden layer *h*_1_, $b_{j}\;$ is the bias associated with the hidden neuron *j*, $w_{ij}\;$ represents the connection weight between input neuron *i* and the hidden neuron *j*, and $x_{i}$ is the input feature *i*. The hidden layers are responsible for identifying the underlying data distribution. Subsequently, these data are assigned to one of the classes, set by a node of the output layer.

The learning process of neural networks is through iterations. In this sense, a data set is fed several times into the algorithm, where the algorithm learns to recognize differences between the training data by adjusting the bias and the weights. Learning the neural network allows for adapting and modifying the weight parameter (*w*) of the threshold. Consequently, the algorithm automatically learns the coefficients of the optimal weights.

#### 2.4.3. Support Vector Machine

The supervised SVM algorithm allows for classifying data in a binary way. It divides the classes of a sample into two spaces by means of a separating hyperplane. Consequently, the hyperplane allows for maximizing in a new space the minimum distance between data of different classes [26]. Support vectors are subsets of training points that are used in the decision function of the algorithm for better accuracy in high-dimensional spaces.

The input data are two sets of *n* dimensional vectors. To train the SVM algorithm, the hyperplane that maximizes the distance (margin) between the support vectors of each class is identified. In this sense, the best hyperplane is the one that “maximizes the margin” between the classes. According to the kernel parameter selected, the SVM algorithm will be linear or nonlinear. The great advantage of this algorithm is that it makes use of the kernel to increase the vector space of the predictor variables, with the objective of establishing a nonlinear boundary between groups of observations. Considering the logic proposed in the study [27], the function associated with the algorithm is shown in Equation (2).

(2) $$f\left(x\right)=\beta_{0}+\sum_{i=1}^{m}\alpha_{i}\;*\;K(x,\;x_{i})\;\;$$

For each training observation, there are *m* parameters $\alpha_{i},\;i=1,\;\ldots ,\;m$. The parameters $\alpha_{1},\;\ldots ,\alpha_{m}\;$ and $\beta_{0}$, are estimated with the internal product $\left(x_{i},\;x_{\hat{i}}\right)\;$ between all pairs of training observations. The internal product between each of the training points $x_{i}\;$ and the new point *x* is calculated to evaluate the function $f\left(x\right)$. The radial basis kernel function is calculated with Equation (3).

(3) $$K\left(x_{i},\;x_{\hat{i}}\right)=e^{\left(-\gamma \sum_{j=1}^{k}{\left(x_{ij}-x_{\hat{ij}}\right)}^{2}\right)}$$

where γ is a hyperparameter to be adjusted for each SVM.

#### 2.4.4. Particle Swarm Optimization

The PSO was created by James Kennedy and Russel Eberhart in 1995 [28]. It is an algorithm based on the collective behavior of animal populations that exchange information to find an optimal solution to a given problem. The main advantages of this algorithm are that it can handle continuous and categorical variables, run on various computers and processors to increase the search process and handle large data sets [19].

The algorithm uses a population of N particles that move within the problem solution space. The position of each particle represents a feasible solution to the problem. To determine the quality or fitness of a particle, the objective function of the problem is applied to the current position of said particle. The algorithm can be applied to either minimization or maximization problems. If applied to a minimization problem, the best solution for the population is the one corresponding to the particle with the lowest fitness. The algorithm applies an iterative process that updates the positions of the particles and takes as the solution to the problem the best of the solutions found by the population during this process.

The PSO randomly defines the initial position and velocity of each particle $\left(x_{i}\left(0\right),v_{i}\left(0\right),\;for\;i=1,\;\ldots ,N\;\right)$. Subsequently, the initial position of each particle is stored as the local best initial position of the particle $\left({pb}_{i}\left(0\right)=x_{i}(0),\;for\;i=1,\;\ldots ,N\;\right)$. Then the fitness of the current position of each particle is calculated and stored ${(f}_{pi},\;for\;i=1,\;\ldots ,\;N)$. The best particle of the initial population is used to define the global best solution of the problem $\left(gb\left(0\right)\right)$ and its fitness $\left(f_{gb}\right)$. The PSO algorithm updates the position and velocity of each particle at each iteration until it reaches the iterations maximum number or converges. Each iteration updates the position (Equation (4)) and velocity (Equation (5)) of each particle *i.*

(4) $$x_{i}\left(t\right)=x_{i}\left(t-1\right)+v_{i}\left(t\right)$$

(5) $$v_{i}\left(t\right)={wv}_{i}\left(t-1\right)+c_{1}r_{1}\left[{pb}_{i}\left(t-1\right)-x_{i}\left(t-1\right)\right]+c_{2}r_{2}\left[gb\left(t-1\right)-x_{i}\left(t-1\right)\right]$$

where $r_{1}\;$ and $r_{2}\;$ are randomly distributed values in the interval [0, 1], *w* is the inertia coefficient, with 0<i<1. The parameters $c_{1}\;$ y $c_{2}$ are acceleration constants, $c_{1}$ determines the effect of the particle’s previous experience in its motion, while $c_{2}$ determines the effect of the swarm’s previous experience. Finally, the fitness function of the new position associated with each particle is calculated and the local best and global best position are updated.

In this study, the hyperparameters used for PSO were determined through an iterative process based on practical experimentation, aiming to optimize model performance. A population size of 50 particles was defined, with an inertia coefficient (w) set at 0.6 to achieve an effective balance between exploration and exploitation of the solution space. The acceleration factors ($c_{1}$) and ($c_{2}$) were set at 2.0. The algorithm ran for a maximum of 100 iterations, ensuring convergence to optimal configurations. The fitness function for each model was designed to maximize key metrics such as AUC, accuracy, recall and F1-score. These values were adjusted considering previous configurations in the literature [28] and specific tests on the dataset, ensuring a suitable balance between computational time and the precision of the optimized models

### 2.5. Validation and Performance Metrics

The *k*-fold cross-validation method was used in the development of the models. This method divides the sample data randomly into partitions with the same size. The value of *k* is the number of partitions into which the sample is divided, in this thing *k* = 10, following the methodology applied in the previous study. Therefore, the dataset is divided into 10 partitions. The model is trained using *k*-1 subsets and the remainder is used for test data. This procedure is replicated 10 times until all subsets have served as the validation set. Its final estimated value will be the average of the values obtained in the *k* iterations.

Model performance metrics are derived from the number of correctly and erroneously classified subjects. In this sense, the confusion matrix is used, which is a tool for comparing predicted values with actual values. The parameters of the confusion matrix are described below:

- True positives (*TP*): Subject’s number with readmission that has been correctly classified;
- False positives (*FP*): Positive subjects’ number (readmission) that have been erroneously classified as a non-readmission;
- True negatives (*TN*): Non-readmission subjects’ numbers that have been correctly classified;
- False negatives (*FN*): Negative subjects’ number (non-readmission) that have been erroneously classified as a readmission.

Based on these parameters, the following model performance metrics were calculated in this study:

- *Accuracy*: This is the proportion of subjects correctly classified by the model (Equation (6));

(6) $$Acc=\frac{TP+TN}{TP+TN+FP+FN}$$

- *Precision*: The proportion between the number of positive results correctly identified and the total number of positive items (readmissions) (Equation (7));

(7) $$Precision=\frac{TP}{TP+FP}$$

- *Sensitivity* or *recall*: The proportion of subjects with readmissions classified correctly (Equation (8));

(8) $$Recall=\frac{TP}{TP+FN}$$

- *Specificity*: This is the proportion of subjects without readmissions classified correctly (Equation (9));

(9) $$Specificity=\frac{TN}{TN+FP}$$

- *F1-score*: It is the harmonic mean between *recall* and *Precision* (Equation (10));

(10) $$F1_{score}=2\;*\;\frac{\left(Precision\;*\;Recall\right)}{Precision+Recall}$$

- *AUC*: The Area Under the Curve (*AUC*) is the overall precision of a sensitivity test versus specificity as determined by a *ROC* curve [29]. The *AUC* can take values between 0 and 1, an *AUC* value of 0.5 means that the test has no discriminatory ability. When it takes values above 0.5, it is considered that the *AUC* can discriminate whether the patient is readmitted or not [30].

## 3. Results Analysis

### 3.1. Database

For the analysis of the study, 6089 hospital admission records corresponding to 3065 patients with schizophrenia from the 11 public hospitals of Castilla and León were included. The demographic data analysis reveals significant gender differences (See Table S1). In this sense, men represent 67.21% (2060 of 3065 patients) and women 32.79% (1005 of 3065 patients), showing that men are the most affected by this psychiatric disorder. In terms of age, the 31 to 50 (*p* = 0.0171) age group is the most representative, with 53.51% and 49.18% in the readmission and non-readmission groups, respectively. For hospitalization data, the mean length of stay among readmitted patients was 18 days (*SD* = 15.361), with a readmission rate of 76.12%. The highest incidence of patients with readmission was recorded in the care complexes of Burgos (317 patients, *p* < 0.0001), Bierzo Hospital (191 patients, *p* = 0.0001), and León (196 patients, *p* = 0.0002).

The variables length of stay (*p* < 0.0001), age (*p* < 0.0001), and hospital (*p* < 0.0001) are significant for the data set, while the variable gender (*p* = 0.1659) shows no significant differences. Clinically, the significant comorbidities of readmission patients in this data set are diagnoses with V-codes (78.15%, *p* < 0.0001), mental disorders other than the main diagnosis (69.77%, *p* < 0.0001), and substance abuse (50.71%, *p* < 0.0001). The more prevalent pathologies in the sample of readmitted patients are continuous cannabis abuse (10.42%, *p* < 0.0001), alcohol abuse (10.29%, *p* < 0.0001), tobacco abuse disorder (16.05%, *p* < 0.0001), personality disorder (2.61%, *p* < 0.0001), delusional disorder (2.74%, *p* = 0.0004) and psychosis (3.37%, *p* < 0.0001). In addition, there are social factors that are collected in the variable Diagnosis Codes V that influence patient readmission. These factors are family history of psychiatric illness (15.62%, *p* < 0.0001), person living alone (5.31%, *p* < 0.0001), and history of non-compliance with medical treatment (21.05%, *p* < 0.0001).

### 3.2. Model Optimization

Considering the main objective of this research and the results obtained in the study [4], a series of models have been optimized to predict the readmission risk of hospitalized patients with schizophrenia in Castilla and León. The Python programming language version 3.11.5 was used for the development and validation of the new optimized ML models. For training and testing, the data set was randomly split in 80/20, respectively. The results obtained in the previous study are shown in Table 1.

Assuming that the research data are of an administrative nature and are not based on the patient’s clinical data, the decision tree-based algorithms improve the performance metrics of the developed models, as shown in Table 1. Algorithms such as Random Forest are particularly effective for handling high-dimensional data, noise, and missing values. They offer advantages in terms of robust classification capabilities without requiring extensive data normalization. Optimization algorithms such as PSO allow performing a search within a group of particles representing possible solutions and obtaining the optimal solution for that data set. Consequently, among the six predictive models proposed in the previous study [4], RF was chosen to be optimized using PSO since RF shows the highest values in the performance metrics evaluated. Also, the SVM and MLP algorithms that show the lowest performance values have been selected.

In this sense, the application of the PSO algorithm to predict the risk of readmission in patients with schizophrenia allows for the optimization of essential hyperparameters for each model, adjusting specific configurations within each algorithm. Consequently, the model’s ability to capture complex patterns in the data was improved, maximizing performance metrics as shown in Table 2. Validation was performed using k-fold cross-validation (k = 10), ensuring the generalizability of the results and minimizing the risk of overfitting. This approach enhances predictive accuracy and demonstrates the feasibility of integrating metaheuristic techniques with ML models to address complex clinical problems.

#### 3.2.1. Random Forest with Particle Swarm Optimization

**Figure 1:**
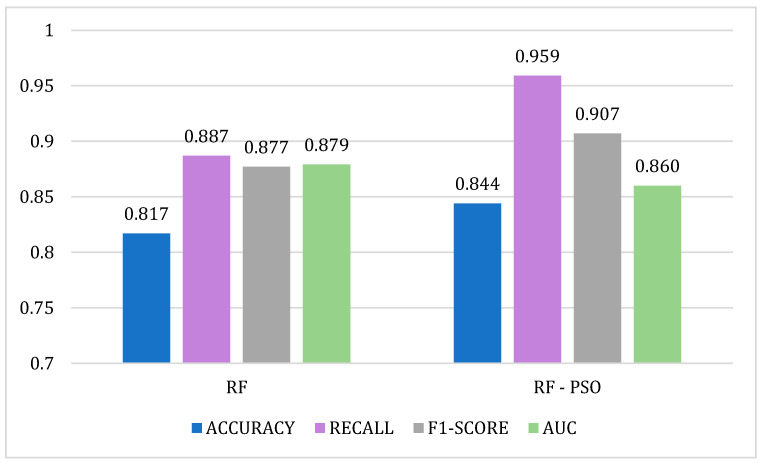
Comparison of performance metrics for the RF and RF-PSO algorithms.

In terms of performance, for the data set used in the study, the RF algorithm was the best classifier for the readmission of patients with schizophrenia. In this sense, we applied PSO to optimize the RF predictive model. A comparison of the performance metrics of the developed RF and RF-PSO algorithms is shown in Figure 1.

The optimal hyperparameters obtained from the fitness function in the PSO are *n_estimators* = 82, *max_depth* = 33, used to train the RF model. The results show an increase in the *accuracy*, *F1-score*, and *recall* metrics, while in the *AUC* metric, the values do not improve (See Figure 1). The percentage improvement between the algorithms for each of the performance metrics is 2.7% for *accuracy*, 7.2% for *recall*, and 3.0% for *F1-score*. These results, in comparison with the study [19], show significantly higher improvement percentages. In [19], the authors develop a clinical monitoring support system for readmission to intensive care units based on PSO and ML algorithms. The comparison between the RF and RF-PSO algorithms shows an improvement of 2.54% for *accuracy* and *recall* and 2.64% for the *F1-score*, while no improvement is shown for the SVM algorithm. Therefore, the combination of PSO with RF in our study is the model that best predicts the readmission risk of patients with schizophrenia in the region of Castilla and León.

#### 3.2.2. Support Vector Machine with Particle Swarm Optimization

**Figure 2:**
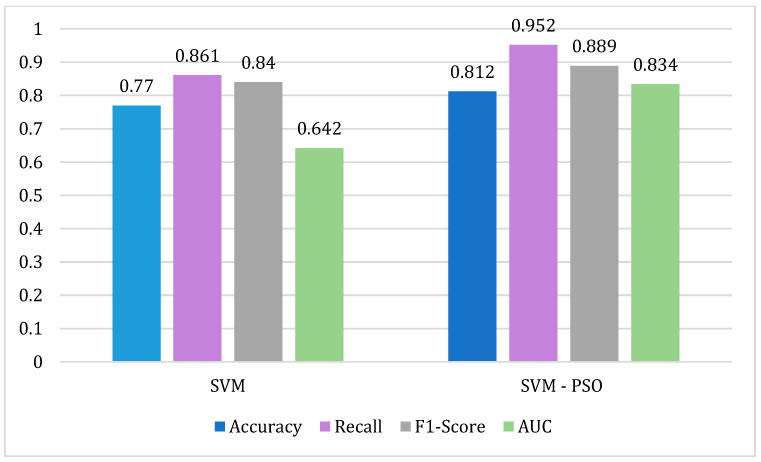
Comparison of performance metrics for the SVM and SVM-PSO algorithms.

The performance metrics of the SVM-PSO model show a significant improvement compared to the SVM model (See Figure 2).

The results show an improvement in all the metrics evaluated. The optimal hyperparameters obtained from the PSO fitness function to train the SVM model are *kernel* = “rbf”, *gamma* = 0.02521672, and *C* = 1.64794017. The percentage improvement between algorithms is 4.2% for *accuracy*, 9.1% for *recall*, 4.9% for *F1-score* and 19.2% for *AUC*. In similar studies, such as [21], the authors develop hospital readmission prediction models combining PSO with ML. The results of this study show a comparison of the SVM and SVM-PSO algorithms, obtaining an improvement of 8.9% for *accuracy* and 18.6% for *recall*. The results of the similar study show significantly higher improvements than those obtained in our study. However, the metric values obtained in our study are higher than those obtained in a similar study.

#### 3.2.3. Multi-Layer Perceptron with Particle Swarm Optimization

**Figure 3:**
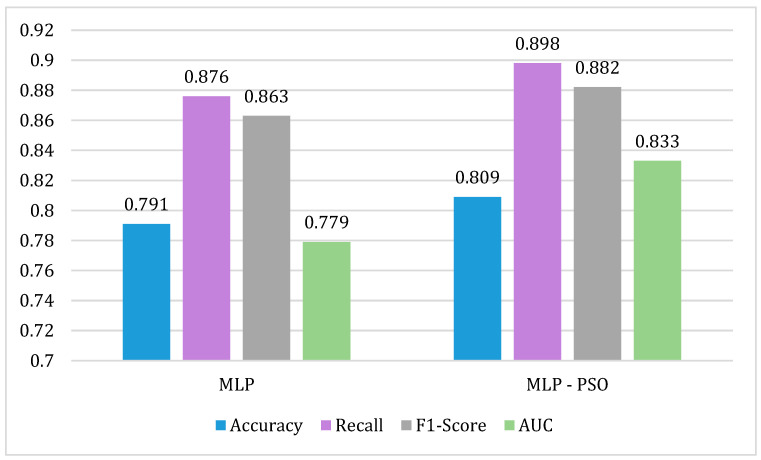
Comparison of performance metrics for the MLP y MLP-PSO algorithms.

The comparison of the performance metrics of the MLP and MLP-PSO algorithms is shown in Figure 3. To train the MLP-PSO model, the optimal hyperparameters obtained from the fitness function in the PSO are *activation* = relu, *alpha* = 0.05, *learning_rate* = adaptive. For each of the metrics, the percentage improvement between the algorithms is 1.8% for *accuracy*, 2.2% for *recall*, 1.9% for *F1-score*, and 5.4% for *AUC* (See Figure 3).

The MLP-PSO model developed is one of the main contributions of this study because no similar studies were found that evaluate the MLP algorithm with PSO in the prediction of hospital readmissions.

### 3.3. Comparative of the Proposed Models

**Figure 4:**
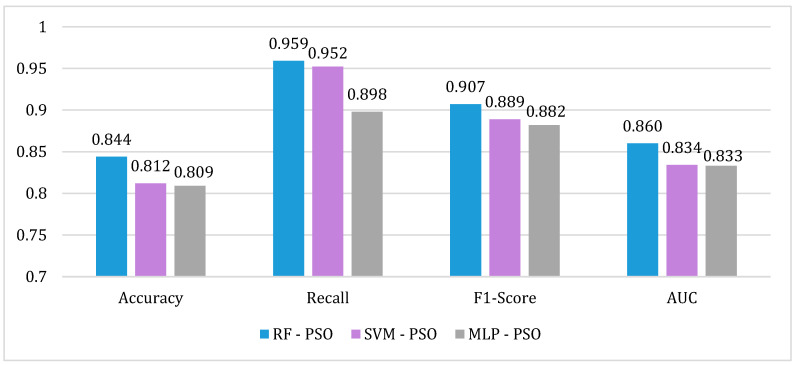
Comparison of performance metrics for the RF-PSO, SVM-PSO, and MLP-PSO algorithms.

In this study, a comparison of the performance of RF, SVM, and MLP models optimized with the PSO algorithm was performed (see Figure 4). With the RF-PSO algorithm, the best prediction values for the risk of readmission of patients with schizophrenia are obtained. In this sense, the values of the performance metrics obtained are an *AUC* = 0.860, *recall* = 0.959, *accuracy* = 0.844, and *F1-score* = 0.907. Considering that the MLP and SVM algorithms obtained the lowest performance values in the previous study [4], optimization with PSO shows a considerable improvement in the results. In this sense, one of the findings of this study is the improvement of the SVM-PSO algorithm with respect to SVM. The optimization of the SVM algorithm achieves the best percentage improvement over the other algorithms, with 4.2% for *accuracy*, 9.1% for *recall*, 4.9% for *F1-score*, and 19.2% for *AUC*.

**Figure 5:**
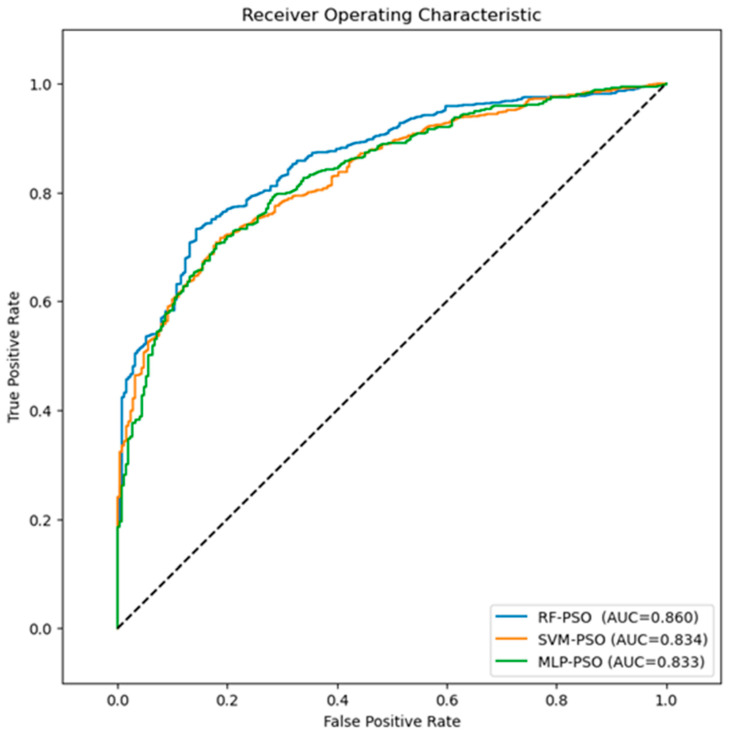
ROC curves for the algorithms proposed in this study.

Figure 5 shows the *ROC* curves of the different algorithms proposed in this study. The results show the RF-PSO algorithm with the highest *AUC* value = 0.860, with respect to the rest of the algorithms. Therefore, the *AUC* and *recall* values of RF-PSO demonstrate that it is the algorithm that best correctly classifies patients who are readmitted to public hospitals in Castilla and León.

## 4. Limitations

The results presented in this research show the improvement of the predictive models using the optimization algorithm PSO compared to the models of the previous study [4]. However, it is necessary to point out some limitations of the research that could have conditioned the results obtained. The data set used only includes administrative data related to patient hospitalization. Consequently, data related to the clinical psychopathology of the patient that could improve the predictive performance of the models were not used. Therefore, the model’s ability to capture specific clinical information, such as symptom severity, comorbidities not reflected in administrative diagnoses, treatment adherence, or deeper social factors, is limited. These variables, which often have a significant impact on clinical outcomes, were unavailable in the dataset and could have enriched the predictive and interpretative capabilities of the models. In future studies, efforts will be made to complement this dataset with clinical information to improve the models’ performance and applicability. Another limitation of the study is that the predictive variables used to develop the models are associated with the risk of readmissions in the population of a specific community in Spain. Therefore, these variables are not generalizable to the rest of the regions, although the methodology used for the development of the models can be applied by other researchers in similar data sets. In addition, it is necessary to mention that only hospitalized patients have been evaluated in the study; it was not possible to include people with schizophrenia who have not been hospitalized.

## 5. Conclusions

In this study, we optimized three models to predict the risk of readmission for patients with schizophrenia in Castilla and León, which have been developed in the previous study [4]. In this sense, we optimized the RF, SVM, and MLP models with the PSO swarm-based algorithm. Comparing each of the optimized models, we obtained that the RF-PSO algorithm demonstrated superior performance across all metrics, achieving an *AUC* = 0.860, *recall* = 0.959, *accuracy* = 0.844, and *F1-score* = 0.907. By optimizing the models, we improved their generalizability and integration in the clinical environment. The results of this study show that predicting the readmission risk for these patients using ML and metaheuristic methods allows the development of preventive strategies. Consequently, preventive strategies help to improve patient well-being and care quality and reduce hospitalization costs in psychiatric services. In this sense, as a future study, we propose analyzing severity levels and mortality risk among hospitalized patients with schizophrenia using optimization and ML algorithms.

## Figures and Tables

**Table 1:** Results of performance metrics from the previous study [4].

Algorithms	AUC	Recall	Accuracy	F1-Score
Random Forest	0.879	0.887	0.817	0.877
SVM	0.642	0.861	0.770	0.840
MLP	0.779	0.876	0.791	0.863

**Table 2:** Results of the performance metrics from this study applying PSO.

Algorithms	AUC	Recall	Accuracy	F1-Score
RF-PSO	0.860	0.959	0.844	0.907
SVM-PSO	0.834	0.952	0.812	0.889
MLP-PSO	0.833	0.898	0.809	0.882

## Data Availability

Third-party data and restrictions apply to the availability of these data. The data were obtained from the Junta de Castilla and León and the Hospital of Zamora; therefore, they are available with the permission of both institutions.

## Supplementary Materials

The following supporting information can be downloaded at: https://www.mdpi.com/article/10.3390/biomimetics9120752/s1, Table S1: Descriptive characteristics of the sample.
