# Supplementary material for: Predictive Modeling of Hospital Readmission of Schizophrenic Patients in a Spanish Region Combining Particle Swarm Optimization and Machine Learning Algorithms
Source: Biomimetics (Basel). 2024 Dec 11;9(12):752. doi: 10.3390/biomimetics9120752 (PMC11673248; doi:10.3390/biomimetics9120752)
Supplement: Supplementary file 1 [file biomimetics-09-00752-s001.zip › biomimetics-3332765-supplementary.pdf]

Table S1. Descriptive characteristics of the sample. Source: Table extracted from previous study (Góngora Alonso et al., 2023).

**Features**	**Readmitted (N=1611 patients)**		**Non-Readmitted (N=1454 patients)**		***p-value***
	**n**	**%**	**n**	**%**	
**Age group**					< 0.0001
< 18 years	5	0.31	5	0.34	
18-30 years	334	20.73	186	12.79	
31-50 years	862	53.51	715	49.18	
51-65 years	302	18.75	374	25.72	
> 65 years	108	6.70	174	11.97	
**Gender**					0.1659
Female	530	32.90	475	32.67	
Male	1081	67.10	979	67.33	
**Hospital**					< 0.0001
Healthcare Complex of Ávila	83	5.15	105	7.22	
Healthcare Complex of Burgos	317	19.68	209	14.37	
Healthcare Complex of León	196	12.17	229	15.75	
Healthcare Complex of Palencia	102	6.33	108	7.43	
Healthcare Complex of Salamanca	185	11.48	195	13.41	
Healthcare Complex of Soria	79	4.90	80	5.50	
Healthcare Complex of Zamora	177	10.99	164	11.28	
Healthcare Complex of Segovia	100	6.21	78	5.37	
University Clinical Hospital of Valladolid	130	8.07	79	5.43	
The Bierzo Hospital	191	11.85	139	9.56	
University Hospital of Rio Hortega	51	3.17	68	4.68	
**Discharge Type**					< 0.0001

Address	1434	89.01	1246	85.69	
Discharge without documented notification	2	0.12	0	0.00	
Transfer to another hospital	100	6.21	155	10.66	
Transfer to medium and long stay centers	5	0.31	4	0.28	
Voluntary discharge	18	1.12	17	1.17	
Other types	52	3.23	32	2.20	
**Schizophrenia Types**					0.1058
Simple schizophrenia	20	1.24	18	1.24	
Disorganized-type schizophrenia	75	4.66	38	2.61	
Catatonic schizophrenia	4	0.25	5	0.34	
Paranoid schizophrenia	1052	65.30	956	65.75	
Latent schizophrenia	6	0.37	3	0.21	
Residual schizophrenia	244	15.15	231	15.89	
Other specified schizophrenia	45	2.79	34	2.34	
Unspecified schizophrenia	165	10.24	169	11.62	
**Secondary diagnosis according to ICD-9 (D2-D16):**					
Mental disorders	1124	69.77	538	37.00	< 0.0001
Substance abuse	817	50.71	518	35.63	< 0.0001
Infectious diseases	111	6.89	64	4.40	0.2403
Endocrine diseases	448	27.81	285	19.60	< 0.0001
Blood diseases	69	4.28	30	2.06	0.3031
Circulatory system diseases	208	12.91	162	11.14	0.0015
Respiratory system diseases	106	6.58	64	4.40	0.0037
Digestive system diseases	153	9.50	82	5.64	0.0021
Genitourinary system diseases	94	5.83	53	3.65	0.0901
Skin and subcutaneous tissue diseases	60	3.72	30	2.06	0.2334

Osteomioarticular system diseases	88	5.46	57	3.92	0.1476
Symptoms, signs, and poorly defined conditions	238	14.77	83	5.71	< 0.0001
Lesions and poisoning	282	17.50	119	8.18	< 0.0016
Diagnosis Codes V	1259	78.15	913	62.79	< 0.0001
**Procedures according to ICD-9:**					
Proc1	1320	81.94	1080	74.28	0.0006
Proc2	875	54.31	573	39.41	< 0.0001
Proc3	419	26.01	267	18.36	0.0198
**Length of Stay (days)**					< 0.0001
Mean (SD)	18 (15.361)		17 (17.183)		
